# Outcomes after Anterior Interosseous Nerve to Ulnar Motor Nerve Transfer

**DOI:** 10.1055/s-0042-1760097

**Published:** 2023-01-11

**Authors:** Jeffrey N. Gross, Steven E. Dawson, Gerald J. Wu, Scott Loewenstein, Gregory H. Borschel, Joshua M. Adkinson

**Affiliations:** 1 Division of Plastic Surgery, Indiana University School of Medicine, Indianapolis, Indiana, United States; 2 Department of Plastic Surgery, Medical College of Wisconsin, Milwaukee, Wisconsin, United States

**Keywords:** nerve transfer, peripheral nerve surgery, ulnar nerve, median nerve, plastic surgery, ulnar nerve trauma, anterior interosseous nerve, deep motor branch ulnar nerve

## Abstract

**Background** Ulnar nerve lesions proximal to the elbow can result in loss of intrinsic muscle function of the hand. The anterior interosseous nerve (AIN) to deep motor branch of the ulnar nerve (DBUN) transfer has been demonstrated to provide intrinsic muscle reinnervation, thereby preventing clawing and improving pinch and grip strength. The purpose of this study was to evaluate the efficacy of the AIN to DBUN transfer in restoring intrinsic muscle function for patients with traumatic ulnar nerve lesions.

**Methods** We performed a prospective, multi-institutional study of outcomes following AIN to DBUN transfer for high ulnar nerve injuries. Twelve patients were identified, nine of which were enrolled in the study. The mean time from injury to surgery was 15 weeks.

**Results** At final follow-up (mean postoperative follow-up 18 months + 15.5), clawing was observed in all nine patients with metacarpophalangeal joint hyperextension of the ring finger averaging 8.9 degrees (+ 10.8) and small finger averaging 14.6 degrees (+ 12.5). Grip strength of the affected hand was 27% of the unaffected extremity. Pinch strength of the affected hand was 29% of the unaffected extremity. None of our patients experienced claw prevention after either end-to-end ( *n* = 4) or end-to-side ( *n* = 5) AIN to DBUN transfer.

**Conclusion** We conclude that, in traumatic high ulnar nerve injuries, the AIN to DBUN transfer does not provide adequate intrinsic muscle reinnervation to prevent clawing and normalize grip and pinch strength.

## Introduction

The ulnar nerve originates from the C8 and T1 nerve roots and travels across the axilla to the medial aspect of the upper arm. It is a mixed motor-sensory nerve that receives sensory afferents from the ulnar aspect of the ring finger, the entire small finger, and ulnar portion of the dorsal hand. It also provides motor innervation to the flexor carpi ulnaris, the ulnar half of the flexor digitorum profundus muscles in the forearm, and most intrinsic hand muscles. 1 Injury to the ulnar nerve leads to clawing of the ulnar digits due to an imbalance between the intrinsic and extrinsic muscles acting on the digits. 2 This presents as metacarpophalangeal (MCP) hyperextension and proximal interphalangeal (PIP) joint flexion. These injuries can be devastating as they affect activities of daily living. 3 In addition to clawing, ulnar nerve injuries commonly result in decreased grip and pinch strength. 4

Although patient presentation and deficits may vary depending on the location of the lesion, 2 studies have shown that high (at or above the elbow) ulnar nerve lesions have worse outcomes than distal lesions. 5 6 7 This is due to the long distance that the motor axons must traverse to reach the motor end plates. Due to the slow regeneration of axons and the distance they must travel, there is progressive loss of the neuromuscular junction motor end plates of the intrinsic muscles of the hand. Surgical options for repair of ulnar nerve injuries have traditionally included nerve grafting and tendon transfers. 8 9 More recently, distal transfer of the anterior interosseus nerve (AIN) to the deep motor branch of the ulnar nerve (DBUN) was proposed. 10 11 12 Distal nerve transfer provides a shorter distance for reinnervation, ultimately reducing time until muscle recovery. Consequently, AIN to DBUN transfer has been recommended to improve grip strength, pinch strength, and functional outcomes. 12 13 14

There is currently a lack of consensus on exactly which outcome measures should be used to assess intrinsic muscle recovery following nerve transfer. For example, McLeod et al assessed postoperative muscle grade using the British Medical Research Council scale (BRMC), 15 whereas Novak and Mackinnon directly evaluated pinch strength, grip strength, and flexion of the MCP joint. 12 Sallam et al evaluated postoperative pinch and grip strength in addition to using a modified BRMC to classify motor and sensory recovery. 13 Others have evaluated claw hand deformity correction by studying MCP joint hyperextension and PIP joint extension. 16 Given the heterogeneity of reported outcomes, the purported benefits of AIN transfer can be difficult to synthesize.

The purpose of our study was to evaluate patient outcomes following AIN to DBUN transfers using clinically relevant outcome measures. We hypothesized that, in traumatic proximal ulnar nerve injuries, AIN to DBUN transfer prevents ulnar claw deformity and improves grip and pinch strength.

## Methods

Following approval by the Indiana University Institutional Review Board, we prospectively studied outcomes from patients who underwent AIN to DBUN transfer following a high ulnar nerve injury from 2016 to 2021 in two level-1 trauma centers in a single city. We included patients undergoing either end-to-end (ETE) or end-to-side (ETS) reconstructions. Enrolled subjects provided written and verbal informed consent to participate

With the exception of nerve coaptation technique (ETS or ETE), the procedures were performed similarly in all patients. We released Guyon's canal and identified the DBUN. We performed internal neurolysis in the distal one-third of the forearm. At this level, the AIN was identified entering the pronator quadratus, dissected distally until branching, and sectioned. Coaptation between the two nerves was performed with 9-0 nylon using the operating microscope (either ETE or ETS) to the DBUN; the repair was reinforced with fibrin glue. All patients were immobilized postoperatively for 3 weeks before initiating therapy.

We measured grip strength with a Jamar Hydraulic Hand Dynamometer. Three separate grip strength measurements were obtained, averaged, and recorded. These measurements were compared to those of the contralateral unaffected hand's average of three readings. We measured the resting MCP joint angle of the ring and small fingers (longitudinal axis of P1= 0) with a goniometer. We measured pinch strength with a Jamar Hydraulic Pinch Gauge as an average of three readings and compared this to the contralateral hand's average of three readings.

Mean and standard deviation were reported for time from injury, postoperative follow-up, MCP joint hyperextension in affected versus unaffected contralateral joints, grip and pinch strength. Standard error of the mean was utilized for grip and pinch strength as measurements were averaged over three readings. Percent of normal grip and pinch strength measurements were stratified by nerve transfer type (ETS or ETE) as well as patient age (≥18 years old).

## Results

Of the 12 patients who underwent AIN to DBUN transfer during the study period, 9 agreed to participate in the study. Mean follow-up time from surgery was 18 months with a range of 2.3 to 46.8 months ( Table 2 ). Patient characteristics are listed in Table 1 . All patients sustained a high ulnar nerve injury from trauma ( Table 1 ).

**Table 1:** Demographics

Patient number	Patient age at procedure (y)	Sex	Mechanism	Laterality
1	13	Female	GSW	Right
2	15	Male	GSW	Left
3	26	Female	GSW	Right
4	28	Female	MVC	Right
5	38	Male	GSW	Left
6	16	Female	MVC	Left
7	33	Male	Stabbing	Left
8	33	Male	Stabbing	Left
9	57	Female	Orthopaedic	Left

Abbreviations: GSW, gunshot wound; MVC, motor vehicle collision.

**Table 2:** Postoperative measurements

Patient	AIN to DBUN transfer type	Time from injury to OR (wk)	Postoperative follow-up (mo)	MCPJ hyperextension affected extremity (Degrees)		MCPJ hyperextension unaffected extremity (Degrees)		Grip strength (lbs)			Pinch strength (lbs)			Froment sign c
				Small	Ring	Small	Ring	Affected	Unaffected	Percent of normal a	Affected	Unaffected	Percent of normal b	
1	ETS	12.1	34.1	25	20	0	0	4	49	8.2	1	11	9.1	−
2	ETS	1.6	6.6	25	30	0	0	40	80	50.0	1	11	9.1	+
3	ETS	43.4	46.8	35	0	5	0	20	70	28.6	7	21	33.3	−
4	ETS	16.6	28.9	5	0	5	0	18	62	29.0	6	14	42.9	−
5	ETS	0.6	2.3	1	5	0	0	21	79	26.6	8	21	38.1	−
6	ETE	29.6	8.7	0	15	5	5	5	60	8.3	1	12	8.3	+
7	ETE	7.1	11.4	20	10	5	0	20	130	15.4	5	24	20.8	−
8	ETE	2.4	3.7	15	0	0	0	42	101	41.6	8	20	40.0	+
9	ETE	21.4	17.2	5	0	0	0	7	22	31.8	7	11	63.6	−
	**Mean**	15.0	17.7	14.6	8.9	2.2	0.6	19.7	72.6	27	4.9	16.1	29	
	**SD/SEM**	14.5	15.5	12.5	10.8	2.6	1.7	8.0 d	17.8 d	14	1.8 d	3.1 d	19	

– Abbreviations: AIN, anterior interosseous nerve; DBUN, deep motor branch of the ulnar nerve; ETE, end-to-end nerve transfer; ETS, end-to-side nerve transfer; MCPJ, metacarpophalangeal joint; SD, standard deviation; SEM, standard error of the mean.

a Percent grip strength of normal (affected/unaffected).

b Percent pinch strength of normal (affected/unaffected).

c Froment's sign – test of adductor pollicis weakness and ulnar nerve palsy by placing paper between patient's index finger and thumb and asking to resist removal of paper. + = Positive sign, patient unable to resist pulling paper. - = negative sign, patient able to resist pulling paper.

d Strength testing sampled over three readings, *n* = 3.

After transfer, clawing was present in the ring and small finger of all patients ( Table 2 ). MCP joint hyperextension of the ring finger averaged 8.9 degrees (± 10.8) and the small finger 14.6 degrees (± 12.5). At last postoperative evaluation, the average grip strength of the injured hand was 27% of the unaffected contralateral side and the average pinch strength was 29%.

When stratified by type of nerve coaptation (ETE or ETS), we did not find a significant difference in grip or pinch strength between groups. Grip strength in ETS versus ETE was 28 versus 24% of normal when compared to the unaffected contralateral extremity. In addition, pinch strength was not statistically different between ETE and ETS ( Table 3 ).

**Table 3:** Stratified grip and pinch strength by nerve transfer type and patient age

	Grip strength percent of normal	Pinch strength percent of normal
Mean (ETS)	28	26
Mean (ETE)	24	26
Mean (pediatric a )	22	9
Mean (adult b )	29	40

Abbreviations: ETE, end-to-end nerve transfer; ETS, end-to-side nerve transfer.

a Pediatric age group defined as patient younger than 18 years old at the time of surgery.

b Adult age group defined as older than 18 years old at the time of surgery.

## Discussion

Early intrinsic muscle reinnervation is the primary goal of the AIN to DBUN transfer. The results of surgery have been assessed with a variety of measurements, including MCP joint hyperextension, PIP joint extensor lag, grip strength, and pinch strength. 4 12 Heterogeneity of outcomes, however, has made interpretation of postoperative outcomes difficult.

Previous studies on AIN to DBUN transfer have primarily focused on the evaluation of motor/sensory recovery after ulnar nerve injury. For instance, Sallam et al evaluated the efficacy of nerve transfer versus nerve graft in the repair of high ulnar nerve injuries using a modified BMRC scale to evaluate motor and sensory recovery, in addition to measuring grip and pinch strength. Of their 24 patients with AIN to DBUN transfers, 12 had M3 motor recovery (contraction against gravity) and 8 patients had M4 motor recovery (contraction against light resistance) when assessing the motor power of ulnarly innervated intrinsics such as the abductor digiti minimi. 13 They found no significant difference in sensory recovery between the nerve transfer and the nerve graft groups. Similar results were reported by Flores who performed a retrospective chart review of patients who underwent nerve grafting verse AIN to DBUN transfer. The MRC M3/M4 outcomes were observed significantly more often in the nerve transfer group (80 vs. 22%), and the mean values for hand grip strength were higher (31.3 ± 5.8 vs. 14.5 ± 7.2 kg) than the nerve grafting group. 14 Chen et al evaluated outcomes in 13 patients who underwent early or late ETS AIN to DBUN transfer for traumatic high ulnar nerve palsy (classified as Sunderland grade IV/V). They found statistically significant motor recovery of ulnar nerve function as assessed by the BMRC score, grip strength, and pinch strength at 6 and 12 months after the injury. Functional recovery was significantly better in the AIN transfer group compared with the control group. More recently, Arami and Bertelli sought to evaluate the effectiveness of AIN to DBUN transfers in prevention of clawing. They quantified clawing by measuring MCP joint hyperextension and PIP extension lag, and ultimately found that none of their 11 patients had improvement in clawing. 16 Our study sought to combine these efforts and evaluate motor recovery in addition to clawing of the hand. Similar to Arami and Bertelli, we found that all patients had persistent digital clawing at last follow up.

Historically, ETE transfers have shown to have superior results when compared to ETS. 4 17 18 19 20 Presumably, these findings result from directing all axonal flow from the donor nerve to the recipient nerve. Conversely, in ETS transfers, the donor nerve must compete against the native recipient nerve to direct axonal input distally. In our study, however, we did not find a significant difference when stratified by type of nerve transfer coaptation.

In our study, the AIN to DBUN transfer did not restore normal intrinsic hand function. While we cannot determine the magnitude of change from preinjury grip or pinch strength, the unaffected contralateral extremity provided an acceptable control. Given these data, we offer a note of caution when counseling patients about their expectations of the effectiveness of the AIN to DBUN in the trauma population with high ulnar nerve injuries.

One possible explanation for inadequate reinnervation of the intrinsic muscles is the differing axon counts in the donor and recipient nerves. Previous cadaveric studies have shown axon ratios of AIN:DBUN to be 1:4.8 where specimens were taken at the hypothetical coaptation site. 21 This significant axon count difference may account for the deficits observed in intrinsic function recovery. 21 Donor nerves with higher axon counts provide greater muscle force and excursion than do nerves with lower axon counts. 22 When the motor innervation of a given muscle falls below 20% of its preinjury level, the muscle will fail to recover its baseline force. 23 In patients undergoing AIN-DBUN nerve transfer, the low number of available donor nerve fibers may be insufficient to restore baseline force.

Limitations of the study include the fact that it was a single academic institution and a small cohort of patients. There was a lack of preinjury data with comparison only to the contralateral unaffected hand, as well as variability of timing between injury and nerve transfer and surgery and follow-up assessment. Further, comparing grip strength and pinch strength to the contralateral hand does not account for hand dominance, which has been shown to affect grip strength. 24 In addition, grip strength and pinch strength can also be affected by functioning median and radial-innervated muscles and this can confound assessments of ulnar-innervated intrinsic muscle function. 4

## Conclusion

In our study, no patient had prevention of clawing after AIN to DBUN transfer. Further, grip strength and pinch strength were significantly lower than the contralateral unaffected hand. Given these findings, we offer a note of caution when counseling patients about the efficacy of AIN to DBUN transfer following high ulnar nerve injuries.
